# Genomic evolution and local epidemiology of *Klebsiella pneumoniae* from a major hospital in Beijing, China, over a 15 year period: dissemination of known and novel high-risk clones

**DOI:** 10.1099/mgen.0.000520

**Published:** 2021-02-25

**Authors:** Mattia Palmieri, Kelly L. Wyres, Caroline Mirande, Zhao Qiang, Ye Liyan, Chen Gang, Herman Goossens, Alex van Belkum, Luo Yan Ping

**Affiliations:** ^1^​ bioMérieux, Data Analytics Unit, La Balme Les Grottes, France; ^2^​ Department of Infectious Diseases, Central Clinical School, Monash University, Melbourne, Victoria, Australia; ^3^​ bioMérieux, R&D Microbiology, La Balme Les Grottes, France; ^4^​ Center for Clinical Laboratory Medicine, First Medical Center of Chinese PLA General Hospital, Beijing, PR China; ^5^​ Laboratory of Medical Microbiology, Vaccine and Infectious Disease Institute, University of Antwerp, Belgium

**Keywords:** *Klebsiella pneumoniae*, Genomics, AMR, hypervirulence, CG258, ST383

## Abstract

*
Klebsiella pneumoniae* is a frequent cause of nosocomial and severe community-acquired infections. Multidrug-resistant (MDR) and hypervirulent (hv) strains represent major threats, and tracking their emergence, evolution and the emerging convergence of MDR and hv traits is of major importance. We employed whole-genome sequencing (WGS) to study the evolution and epidemiology of a large longitudinal collection of clinical *K. pneumoniae* isolates from the H301 hospital in Beijing, China. Overall, the population was highly diverse, although some clones were predominant. Strains belonging to clonal group (CG) 258 were dominant, and represented the majority of carbapenemase-producers. While CG258 strains showed high diversity, one clone, ST11-KL47, represented the majority of isolates, and was highly associated with the KPC-2 carbapenemase and several virulence factors, including a virulence plasmid. The second dominant clone was CG23, which is the major hv clone globally. While it is usually susceptible to multiple antibiotics, we found some isolates harbouring MDR plasmids encoding for ESBLs and carbapenemases. We also reported the local emergence of a recently described high-risk clone, ST383. Conversely to strains belonging to CG258, which are usually associated to KPC-2, ST383 strains seem to readily acquire carbapenemases of different types. Moreover, we found several ST383 strains carrying the hypervirulence plasmid. Overall, we detected about 5 % of simultaneous carriage of AMR genes (ESBLs or carbapenemases) and hypervirulence genes. Tracking the emergence and evolution of such strains, causing severe infections with limited treatment options, is fundamental in order to understand their origin and evolution and to limit their spread. This article contains data hosted by Microreact.

## Data Summary

(1) Genome assemblies were deposited in the NCBI database under the bioproject accession no. PRJNA657553. Single genome accessions are provided in Tables S1 and S2 (available in the online version of this article).

(2) The antimicrobial susceptibility testing results (VITEK2) are provided in Tables S1 and S2.

(3) The accession numbers of the publicly available genomes used for comparative purposes are provided in Tables S3–S5.

The authors confirm all supporting data, code and protocols have been provided within the article or through supplementary data files.

**Impact Statement**

This study aimed to understand the population structure of *K. pneumoniae* within a major hospital in Beijing, China, over a 15 year period. While previous studies have investigated the genetic epidemiology of ESBL- and carbapenemase-producing *K. pneumoniae *, this study represents the first longitudinal investigation focusing on the broad *K. pneumoniae* population from China. Focusing on a broader bacterial population allowed the understanding of the evolution towards MDR and hypervirulence, as well as the convergence of these two traits, and provides essential information for genomic surveillance efforts. We showed that even in a single clinical setting the *K. pneumoniae* population is highly diverse, and we investigated the emergence of MDR-hv hybrid strains, since the surveillance of such strains is extremely important in order to track their evolution and to limit their spread and clinical impact.

## Introduction

*Klebsiella pneumoniae* is one of the greatest threats for public health amongst Gram-negative pathogens. Multidrug-resistant (MDR) strains causing hospital outbreaks and hypervirulent strains causing severe community-acquired infections are those of major concern [1]. In China, hypervirulent *K. pneumoniae* (hvKp), primarily of clonal group (CG) 23, and carbapenem-resistant *K. pneumoniae* (CR-Kp), mostly belonging to CG258, represent the two major clinically significant *K. pneumoniae* pathogens causing community-acquired and healthcare-associated infections, respectively [2, 3].

HvKp infections are characterized by high morbidity and mortality as they are mainly associated with severe life-threatening liver abscesses, pneumonia, meningitis and endophthalmitis that can occur in young and healthy individuals [4]. Several key virulence factors have been reported in hvKp strains. The capsular polysaccharide (cps) is a major virulence factor for all *K. pneumoniae *, but hvKp strains are usually associated with K1 or K2 capsular serotypes that were shown to be particularly antiphagocytic and serum resistant [1, 5]. hvKp also harbours other virulence genes: (i) the *rmpA* and *rmpA2* genes that upregulate capsule expression; and (ii) the yersiniabactin (*ybt*), aerobactin (*iuc*) and salmochelin (*iro*) siderophores that enhance survival in the blood by promoting iron scavenging [1]. The *ybt* locus is mobilized in the *K. pneumoniae* population by an integrative, conjugative element termed ICE*Kp*, and is commonly found among strains causing community-acquired infections as well as those causing healthcare-associated infections [6]. In contrast, the *iro*, *iuc* and *rmpA*/*rmpA2* loci are usually co-harboured by a virulence plasmid [7] and are generally rare among strains causing healthcare-associated infections. CG23 and other hvKp strains are usually susceptible to most antibiotics [8], however the last few years have seen the emergence of MDR strains, including those resistant to carbapenems, termed CR-hvKp [9–13].

Carbapenem resistance is rapidly increasing in China, and the CHINET surveillance network showed that the resistance rate of *K. pneumoniae* to imipenem and meropenem was respectively increased from 3.0 and 2.9 % in 2005–25 and 26.3 % in 2018, with more than eightfold increase [14, 15]. KPC-2 is the most prevalent enzyme among CR-Kp in China, with 77 % KPC-2 positive strains among the carbapenemase producers reported in a recent study [16]. CG258, comprising sequence type (ST) 258, ST11 and close relatives, is recognized worldwide as a major clinical carbapenem-resistant clone and the major vector of KPC-2, with ST258 being prevalent in Europe and the USA [17] and ST11 accounting for 75 % of CR-Kp in China [16]. Genomic studies revealed that most of the ST11 CR-Kp strains in China harbour a capsule of type K47 or the recently emerging K64 [18, 19]. Recently, CR-Kp ST11 strains carrying the hypervirulence-associated *iuc* locus, have emerged [18–25]. The earliest reports detailed sporadic isolations, but in 2017 a fatal outbreak was reported in an intensive care unit in Hangzhou, Zhejiang province of China [20]. Further retrospective investigations revealed that similar strains were already circulating within China before this outbreak [20, 21], and more recent studies suggest that ST11/K47-*iuc*+is now distributed across many provinces [19, 24, 25].

Numerous studies have investigated the genomic epidemiology of CR-Kp or hvKp in China [19, 25, 26], but few have reported holistic investigations of clinical *K. pneumoniae* populations. We here investigated a collection of *K. pneumoniae* strains obtained from the H301 Beijing hospital during the period 2002–2016, including a subset of 200 randomly selected isolates, representing the broader *K. pneumoniae* population. We aimed to employ phenotypic antimicrobial susceptibility testing and whole-genome sequencing (WGS) to study the evolution and local epidemiology of the *K. pneumoniae* strains circulating within the hospital during the study period. Focusing on a broader bacterial population, instead of CR-Kp or hypervirulent infections only, allowed the understanding of the evolution towards MDR, including extended-spectrum β-lactamase (ESBL) production, and hypervirulence, as well as the convergence of these two traits, providing essential information for genomic surveillance efforts.

## Methods

### Bacterial isolates and antimicrobial susceptibilities

Bacterial isolates were obtained from the 4000-bed Hospital 301 in Beijing, China. A total of 300 *K *. *pneumoniae* isolates were collected from routine microbiological cultures of clinical samples (urine, blood, sputum, tissue biopsies, etc) in the period between 2002–2016. One isolate was further identified as *K. michiganensis* and was excluded, leaving 299 isolates. Of those, 200 were randomly selected for epidemiological and statistical purposes, while the others had been selected based on different criteria [multiple isolates from single patients and obtained from different samples (*N*=37), imipenem-resistant isolates (*N*=28), multiple isolates from one patient with a persisting (at least 3 months) infection (*N*=22), isolates from an outbreak (*N*=12), see ‘selection’ column of Table S2] and were included here to enrich the detailed analysis of major clones. Antimicrobial susceptibility testing was performed with the Vitek2 automated system (bioMérieux, Marcy L’Étoile, France), and results were interpreted according to the EUCAST breakpoints [27]. Antimicrobials tested were: amikacin, aztreonam, cefepime, ceftazidime, ciprofloxacin, ertapenem, gentamycin, imipenem, levofloxacin, piperacillin/tazobactam, tobramycin and trimethoprim/sulfamethoxazole. We defined multidrug resistance as non-susceptibility to three or more classes of antimicrobials, as described before [28].

### Whole genome sequencing and assembly

Genomic DNA was extracted with the DNeasy UltraClean kit (Qiagen, Hilden, Germany), quantified by using the Qubit fluorometer (Thermo Fisher Scientific, USA) and quality checked using the 260/280 ratio absorbance parameter as determined by the DS-11 FX + instrument (DeNovix, Wilmington, USA). Multiplexed Nextera XT libraries were sequenced on a HiSeq platform (Illumina, San Diego, USA) and a 2×150 bp paired-end approach. Raw data from paired-end sequencing were quality checked with the FastQC tool (v.0.11.6) and assembled with SPAdes (v.3.11.1) [29]. Assemblies were inspected with Bandage (v0.8.1) [30].

### Genotyping, antimicrobial resistance (AMR) and virulence prediction

Sequence types (STs) were assigned by the mlst tool (github.com/tseemann/mlst) using the *K. pneumoniae* MLST scheme available via the Pasteur database (bigsdb.pasteur.fr/ bigsdb.pasteur.fr/) [31]. The ABRicate tool (github.com/tseemann/abricate) was used to detect acquired antimicrobial resistance genes using the ResFinder database [32], while plasmid replicons were predicted by PlasmidFinder [33]. The genetic background of acquired AMR and virulence genes was investigated with Bandage (v0.8.1) [30]. Kaptive was used for the capsular-type detection [34]. Kleborate (v0.4.0-b)(github.com/katholt/Kleborate) was used for the species identification, detection of ICE*Kp* associated virulence loci [yersiniabactin (*ybt*), colibactin (*clb*)], virulence plasmid associated loci [salmochelin (*iro*), aerobactin (*iuc*), hypermucoidy (*rmpA*, *rmpA2*)] and for checking the *ompK35/36* genes integrity.

Statistical analyses were performed with Python (v3.7.6) and SciPy (1.4.1) by using a linear model.

### Phylogeny and core SNPs analysis

Phylogenetic analyses of CG258, CG23 and ST383 were performed separately by using the reference genomes GD4 (accession no. CP025951), SGH10 (CP025080) and KpvST383_NDM_OXA-48 (CP034200), respectively. The genomes from each ST/CG were mapped against their respective reference genome by using Snippy (https://github.com/tseemann/snippy). Publicly available genomes were also included for comparative purposes (CG258, *N*=479; CG23, *N*=47; ST383, *N*=10) (Tables S3–S5). For CG258, the phylogeny focused on STs, which are abundant in China, therefore we mostly included publicly available genomes of ST11 and excluded those of ST258. The whole-genome alignments obtained were screened for recombination using Gubbins (v2.3.4) [35], and the recombination-free alignments (length: CG258, 2698 bp; CG23, 3914 bp, ST383, 1046 bp) were used for ML phylogenies by using RAxML (v8.2.12) [36] with the GTRGAMMA model and 100 bootstrap replicates. Core-genome SNPs were obtained with the snp-dists tool (github.com/tseemann/snp-dists) applied to the Gubbins output. Phylogenetic trees were visualized together with associated metadata using Microreact (v7.0.0) [37], Phandango [38] or iTol [39]. The Harvest suite was used to align and visualize genomes of CG23 and ST35 in order to decipher the recombination events within ST1265 [40].

## Results

A total of 299 *K *. *pneumoniae* strains were successfully sequenced. Of those, 200 were randomly selected over the 15 year period (2002–2016), and the following epidemiological investigations will be limited to these 200 strains, unless differently stated. Whole-genome-sequencing data of the 299 clinical isolates have been deposited under BioProject PRJNA657553. Isolate information, genotyping results and individual genome accession numbers are shown in Tables S1 and S2.

### Antimicrobial susceptibility phenotypes

**Fig. 1.**
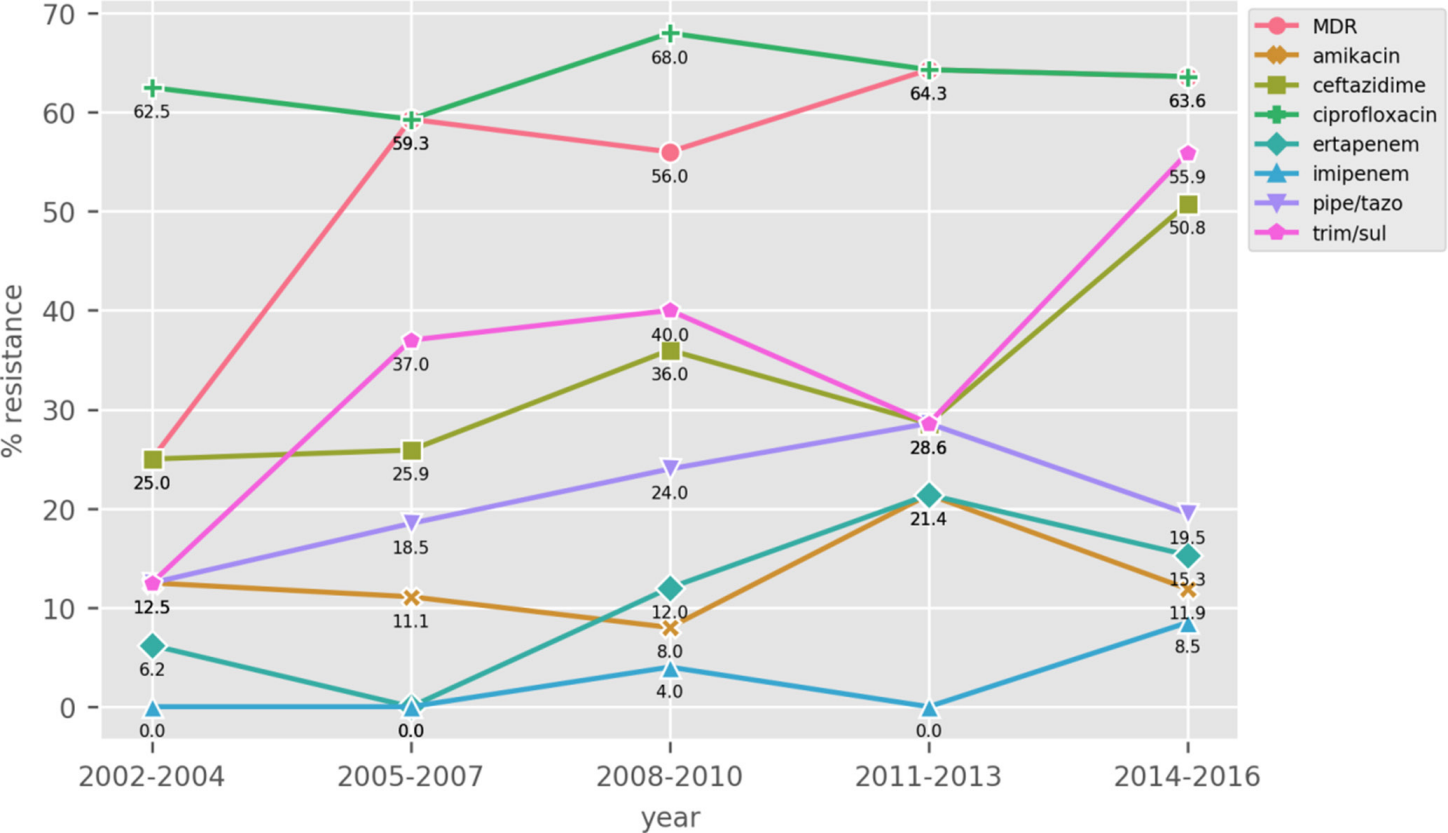
Resistance trends over the three 5 year periods of some selected antibiotics. The trend of the MDR prevalence is also shown.

Phenotypic results showed that the majority of the isolates were classified as MDR (*N*=118, 59 %). Overall, imipenem was the most effective drug, showing an average resistance rate of 5.5 %, followed by amikacin and ertapenem (12.5 % average resistance for both antibiotics) (Table 1). Fig. 1 shows that the resistance rates were fairly stable over the study period for most of the antibiotics. A linear regression analysis showed that only imipenem (*P*-value=0.024) and ceftazidime (0.045) had statistically significant increasing trends with progressing of the years.

**Table 1.** Percentages of resistance over the 3 year periods and total average. The trend of the MDR prevalence is also shown. A linear regression model was used to test whether the changes in resistance rates were statistically significant, and the resulting *P*-values are included in the table

Antibiotic	2002–2004 (*N*=16)	2005–2007 (*N*=27)	2008–2010 (*N*=25)	2011–2013 (*N*=14)	2014–2016 (*N*=118)	Total (*N*=200)	*P*-value
**amikacin**	12.5	11.1	8.0	21.4	11.9	12.5	0.704
**aztreonam**	37.5	40.7	44.0	42.9	54.2	49.0	0.495
**cefepime**	12.5	29.6	16.0	14.3	28.8	25.0	0.197
**ceftazidime**	25.0	25.9	36.0	28.6	50.8	42.0	0.045
**ciprofloxacin**	62.5	59.3	68.0	64.3	63.6	63.8	0.614
**ertapenem**	6.2	0.0	12.0	21.4	15.3	12.5	0.058
**gentamycin**	18.8	33.3	44.0	35.7	46.6	42.1	0.207
**imipenem**	0.0	0.0	4.0	0.0	8.5	5.5	0.024
**levofloxacin**	37.5	33.3	36.0	21.4	39.0	36.9	0.663
**pipe/tazo**	12.5	18.5	24.0	28.6	19.5	20.2	0.222
**tobramycin**	18.8	44.4	56.0	57.1	47.5	46.7	0.149
**trim/sul**	12.5	37.0	40.0	28.6	55.9	46.2	0.223
**MDR**	25.0	59.2	56.0	64.3	63.6	59.0	0.261

### Species and clonal diversity

*In silico* species identification reported the presence of the four major taxa in the *K. pneumoniae* species complex, which are known to be difficult to distinguish by standard clinical laboratory diagnostic techniques: *K. pneumoniae sensu stricto* was the most common (*N*=177, 88.5 %) followed by *K. quasipneumoniae* subsp. *similipneumoniae* (*N*=11, 5.5 %), *K. quasipneumoniae* subsp. *quasipneumoniae* (*N*=8, 4 %) and *K. variicola* subsp. *variicola* (*N*=4, 2 %).

A total of 98 different sequence types (STs) were observed, including 27 novel STs. The majority of STs (72.4 %) were represented by only one strain, highlighting the diversity within the *K. pneumoniae* population. Eight clonal groups were represented by at least five strains, including the globally distributed MDR clones, CG258 (*N*=28), CG15 (*N*=18), CG37 (*N*=13), CG147 (*N*=8) and CG307 (*N*=7), as well as the common hypervirulent clones, CG23 (*N*=14) and CG65 (*N*=9).

A total of 73 different capsule (K) loci were detected, with 60 of them represented by a maximum of three isolates each. The major K loci were KL2 (associated with capsule type K2, *N*=21, including ST14, CG65, ST380, ST86 and ST25), KL1 (associated with capsule type K1, *N*=17, including ST23, ST367 and two novel STs), KL47 (associated with capsule type K47, *N*=9, strictly linked to ST11) and KL102 (capsule type not serologically defined, *N*=9, including ST307, ST20 and ST45). CG258 strains had the highest number of K loci, with 12 distinct loci detected, of which 11 were detected among ST11 strains. CG37 was the second clonal group by K locus diversity, with eight different loci detected. As expected, CG23 and CG65, the hypervirulent clones, were associated with only a single K locus each; KL1 and KL2, respectively [41].

### AMR determinants

**Fig. 2.**
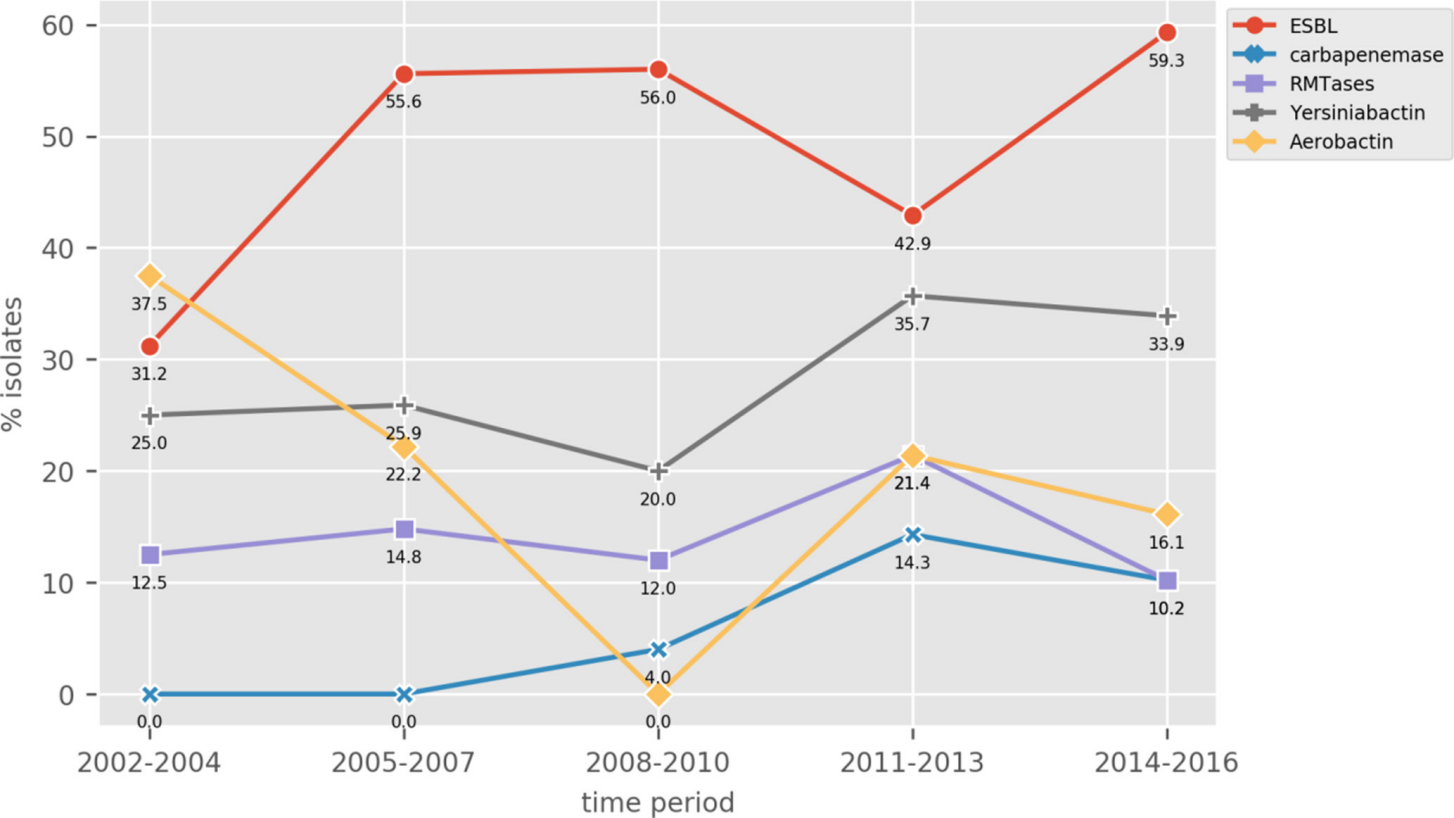
Trends of selected AMR and virulence gene presence percentages.

**Fig. 3.**
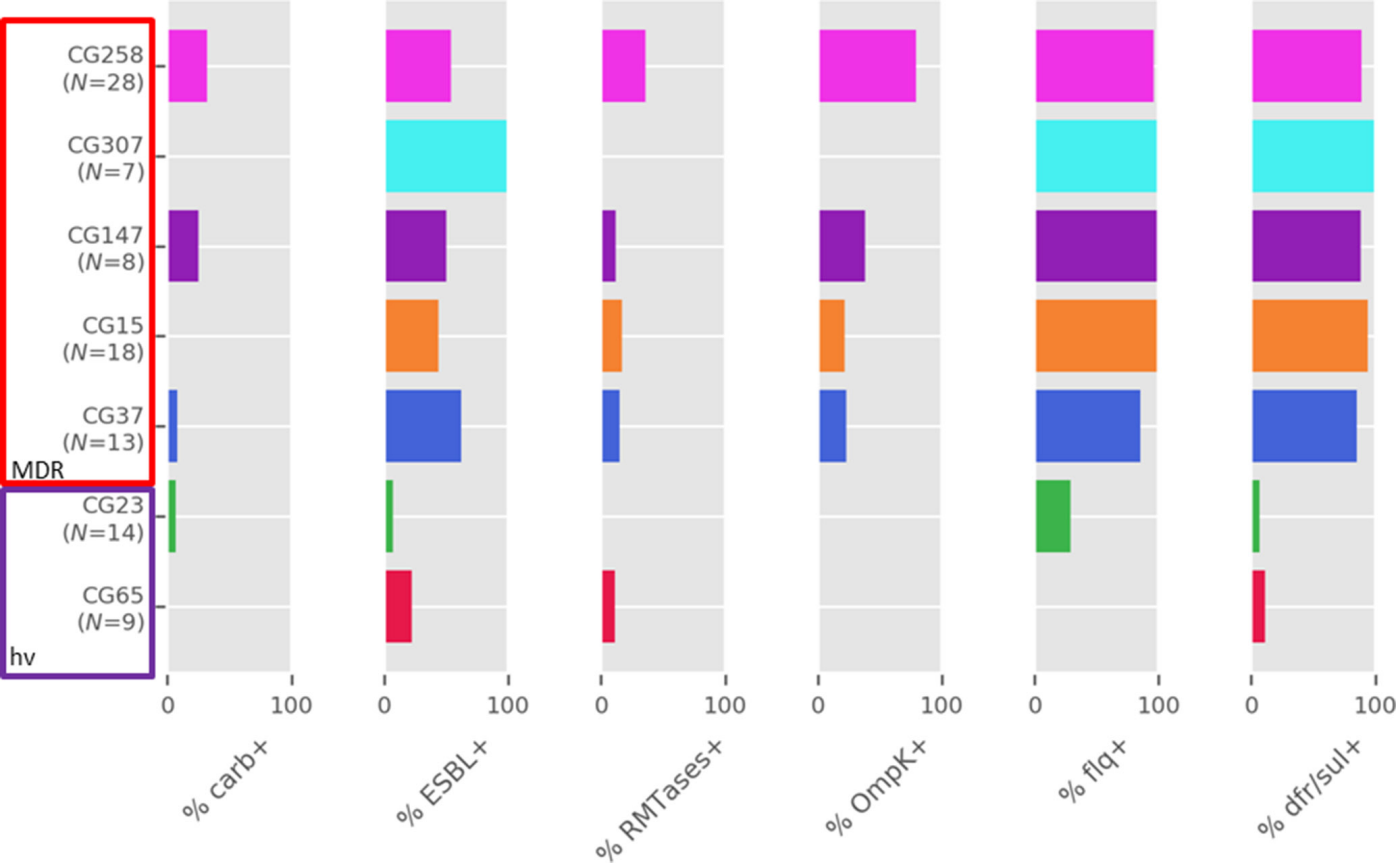
Prevalence of AMR determinants over major CGs. carb: carbapenemase; ESBL: extended-spectrum β-lactamase; RMTases: 16S-RMTases (*armA*, *rmtB*); OmpK: OmpK35/36 alterations; flq: genes or mutations associated to fluoroquinolones resistance; dfr/sul: carriage of *dfr* or *sul* genes associated with resistance to trimethoprim and sulphonamide resistance, respectively.

Overall, more than half of the isolates (*N*=110, 55 %) harboured an ESBL-encoding gene, with a slight increase in prevalence over time (Fig. 2). The most common ESBLs observed were of the CTX-M type, with CTX-M-14 (*N*=35), CTX-M-3 (*N*=26) and CTX-M-15 (*N*=22) being the most prevalent. CG307 strains had the highest prevalence of ESBLs among the common clones, with all strains encoding for either CTX-M-15 or CTX-M-14 (Fig. 3).

Four different carbapenemase-encoding genes were observed, *bla*_KPC-2_ (*N*=10), *bla*_IMP-4_ (*N*=2), *bla*_OXA-48_ (*N*=2) and *bla*_IMP-30_ (*N*=1). No carbapenemase-encoding genes were detected in the period 2002–2006, while two (4.6 %) and 13 (10.2 %) were detected over the 2007–2011 and 2012–2016 periods, respectively. Strains belonging to CG258 represented 60 % of the carbapenemase producers. CG258 strains also had the highest rate of OmpK alterations which are associated with increased carbapenem MICs [42], reaching 78.6 %. OmpK alterations were observed in 42 strains overall (21 %) and resulted in premature termination of OmpK35, in a few cases (*N*=9) combined with additional OmpK36 alterations. There was no difference in the prevalence of OmpK alterations between the 5 year periods.

Genes encoding 16S ribosomal RNA methyltransferase (16S-RMTase) were observed (*N*=24, 12%), with 13 strains harbouring *armA*, 11 harbouring *rmtB* genes and two strains harbouring both *armA* and *rmtB*. Such genes were mainly observed in strains belonging to ST11 (*N*=9) and ST15 (*N*=4). The prevalence of 16S-RMTases genes did not change over time (Fig. 2).

Fluoroquinolones (*aac(6′)-Ib-cr* and *qnr* genes, *gyrA* and *parC* mutations) and trimethoprim-sulfamethoxazole (*dfr*, *sul*) resistance markers were abundant and were observed also within strains belonging to normally antimicrobial susceptible CGs, such as CG23 (28.6 and 7.1% prevalence for fluoroquinolones and trimethoprim/sulfamethoxazole resistance mechanisms, respectively) (Fig. 3).

Acquired mechanisms of colistin resistance were also observed. The *mcr-1.1* gene was observed in the *K. pneumoniae* ST231 strain K089 isolated in 2015. Manual inspection of the assembly graph with Bandage revealed that this gene was carried by a plasmid with replicon IncX4 and identical to plasmid pMCR_WCHEC1618 (accession no. KY463454.1) obtained from an *E. coli* strain from China in 2015 [43]. Strain K089 also carried the gene encoding ESBL CTX-M-27, as well as fluoroquinolones, trimethoprim and sulfonamides resistance markers. Two *mcr-9.1* genes were detected in *K. quasipneumoniae* subsp. *quasipneumoniae* K7029 and K7030 strains belonging to ST1681 and collected in 2005. Unfortunately, we were not able to determine the genetic context of the *mcr-9.1* genes due to the complex nature of the relevant assembly graph regions.

### Acquired virulence determinants

In our collection, yersiniabactin-encoding genes (*ybt*) were observed in 61 strains (30.5 %), and were associated with eight different ICE*Kp* chromosomally integrated mobile elements and one plasmid. The major mobile elements were ICE*Kp10* (*N*=22) and ICE*Kp3* (*N*=17). While ICE*Kp10*, which also carries the colibactin genotoxin locus, *clb*, was linked to hypervirulent clones (CG23, *N*=14; CG65, *N*=5), ICE*Kp3* was most associated with CG258 (*N*=9) and other non-hypervirulent clones. By considering the whole collection excluding duplicates and closely related isolates from outbreaks, we detected *ybt* genes in 62.5 % (25/40) and 100 % (17/17) for CG258 and CG23 isolates, respectively.

**Fig. 4.**
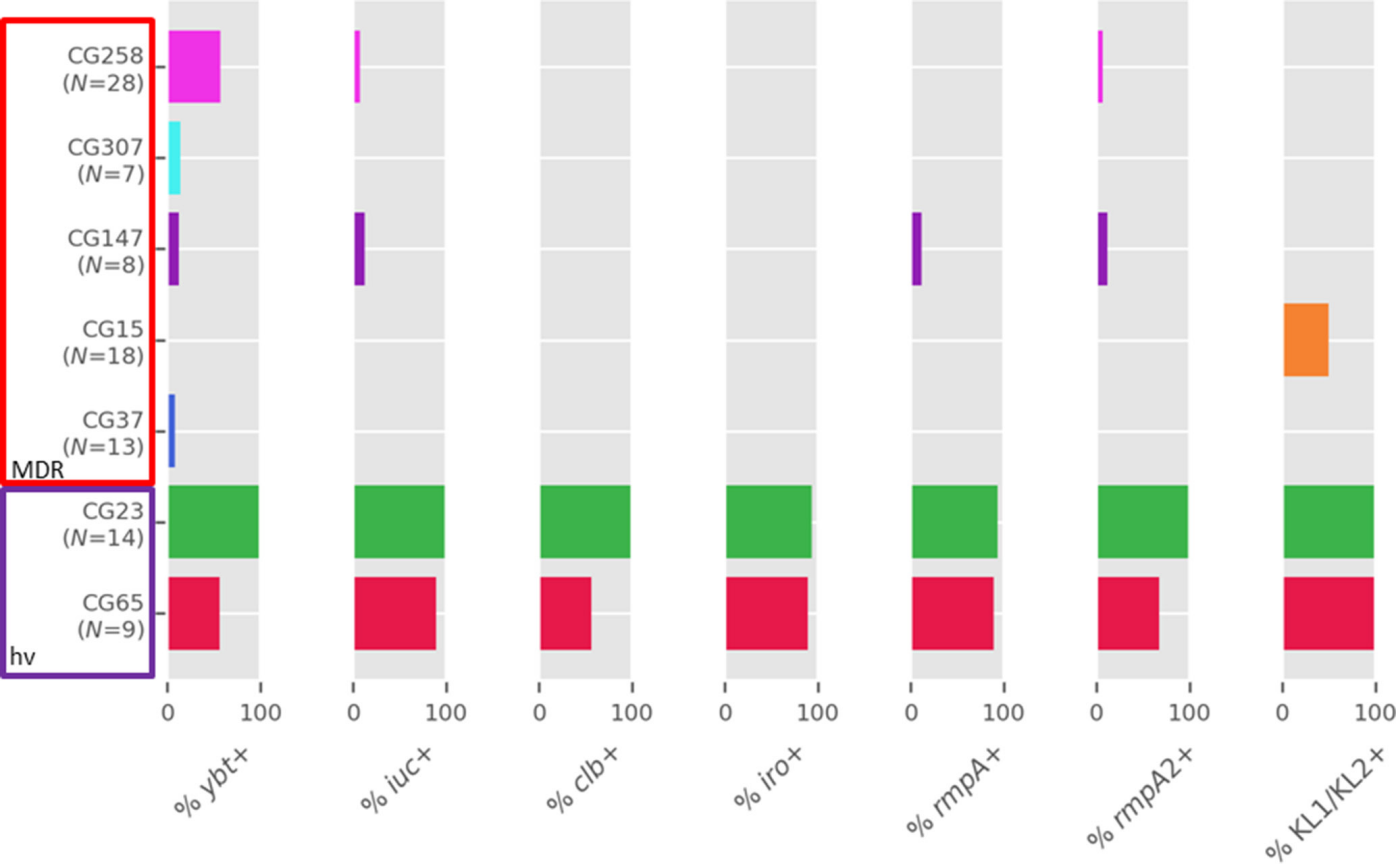
Virulence genes and KL1/KL2 cps locus prevalence within the major CGs.

The plasmid-associated *iuc*, *iro*, *rmpA* and *rmpA2* genes were also observed (*iuc*, 17 %; *iro*, 16.5 %; *rmpA*, 16 %; *rmpA2*, 15 %; *clb*, 11 %), mostly associated with CG23 (100 % prevalence) and CG65 (88.9 %) (Fig. 4). *iuc* lineage 1 (*iuc1*) was the most prevalent *iuc* locus (*N*=32), and was linked to CG23 (*N*=14), CG65 (*N*=8) and six other less represented CGs, including non-hvKp clones and including a *K. quasipneumoniae* subsp. *similipneumoniae* strain. *iuc1* is usually located within the KpVP-1 virulence plasmid (pLVPK and pK2044-like plasmids) [7] together with the previously mentioned virulence genes. We found *iuc1* together with *iro1* (*N*=28), *rmpA* (*N*=28) and *rmpA2* (*N*=29). Other *iuc* lineages observed were *iuc2*, which is associated to KpVP-2 (Kp52.145pII-like) [7] and was observed in an ST380 strain, and *iuc5*, observed in an ST107 strain.

While we observed a slight increase of *ybt* prevalence over time, we did not observe any particular trend for the plasmid-associated virulence genes (Fig. 2).

### Simultaneous carriage of acquired AMR and hypervirulence-associated genes

We detected 11 cases of genotypic convergence of virulence and MDR – indicated by the presence of the aerobactin locus (*iuc*) plus either an ESBL- or a carbapenemase-encoding gene – in our 200 randomly selected strains (5.5 %). An additional 14 convergent isolates were identified from the broader sample collection.

We detected both AMR determinants acquisition by previously described hv STs (*N*=7, 28 %) and virulence plasmid acquisition by previously described MDR STs (*N*=18, 72 %). Most cases of convergence (*N*=21, 84 %) were characterized by the presence of a pLVPK-like plasmid carrying *iuc1,* and occasionally *iro1* (8/21), *rmpA* (14/21) and *rmpA2* (21/21). The remaining convergent strains carried *iuc3* (*N*=3) and *iuc5* (*N*=1).

All convergent strains belonged to *K. pneumoniae sensu strictu*, except strain K898, which belonged to *K. quasipneumoniae* subsp. *similipneumoniae *. Strain K898 belonged to ST367, had K locus KL1 and carried a pLVPK-like plasmid containing *iuc1*, *iro1*, *rmpA* and *rmpA2* and an IncFII plasmid containing the *bla*_CTX-M-15_ gene.

Convergent *K. pneumoniae sensu strictu* isolates belonged to 14 different STs, with major CGs including CG42 (ST383, *N*=6), CG23 (ST23, *N*=3; ST1265, *N*=1) and CG258 (ST11, *N*=3). These CGs will be discussed further below.

### Comparative genomics of CG258 strains: capsule diversity and hypervirulence

Following the epidemiological investigation of our randomly sampled isolate collection we sought to explore the three common clones in further detail. Our broader collection of 299 genomes (random sample plus 99 additional isolates selected for sequencing for various reasons as described in Methods) contained a total of 46 non-duplicated CG258 genomes, including six different STs (ST11, *N*=39; ST11-1LV, *N*=3; ST437, ST1264, ST340, ST1326, *N*=1 each). Seventeen different K loci were detected overall, 11 among ST11 strains. The major K locus was KL47 (*N*=19, 41.3 %), which was first detected in 2014 and then became dominant, accounting for 73.1 % of CG258 strains collected from 2014 onwards.

The prevalence of carbapenemase-encoding genes among our CG258 genomes was 45.7 % (*N*=21), and *bla*_KPC-2_ was the only gene detected. The *bla*_KPC-2_ gene was strongly associated with ST11/KL47 Kp compared to ST11 Kp having other K loci (detected in 100 % of KL47 vs. 7.4 % for other K loci combined; *P*<1×10^−6^; Fisher’s exact test). Yersiniabactin prevalence within CG258 accounted for 62.5%, which was significantly higher than previously reported for this clonal group [odds ratio (OR)=2.6, *P*=0.0046, Fisher’s exact test] [44]. In particular, yersiniabactin prevalence was higher for ST11/KL47 Kp strains, being 100 % for KL47 vs. 44.4 % for other K loci combined (*P*=5.3×10^−5^; Fisher’s exact test).

**Fig. 5.**
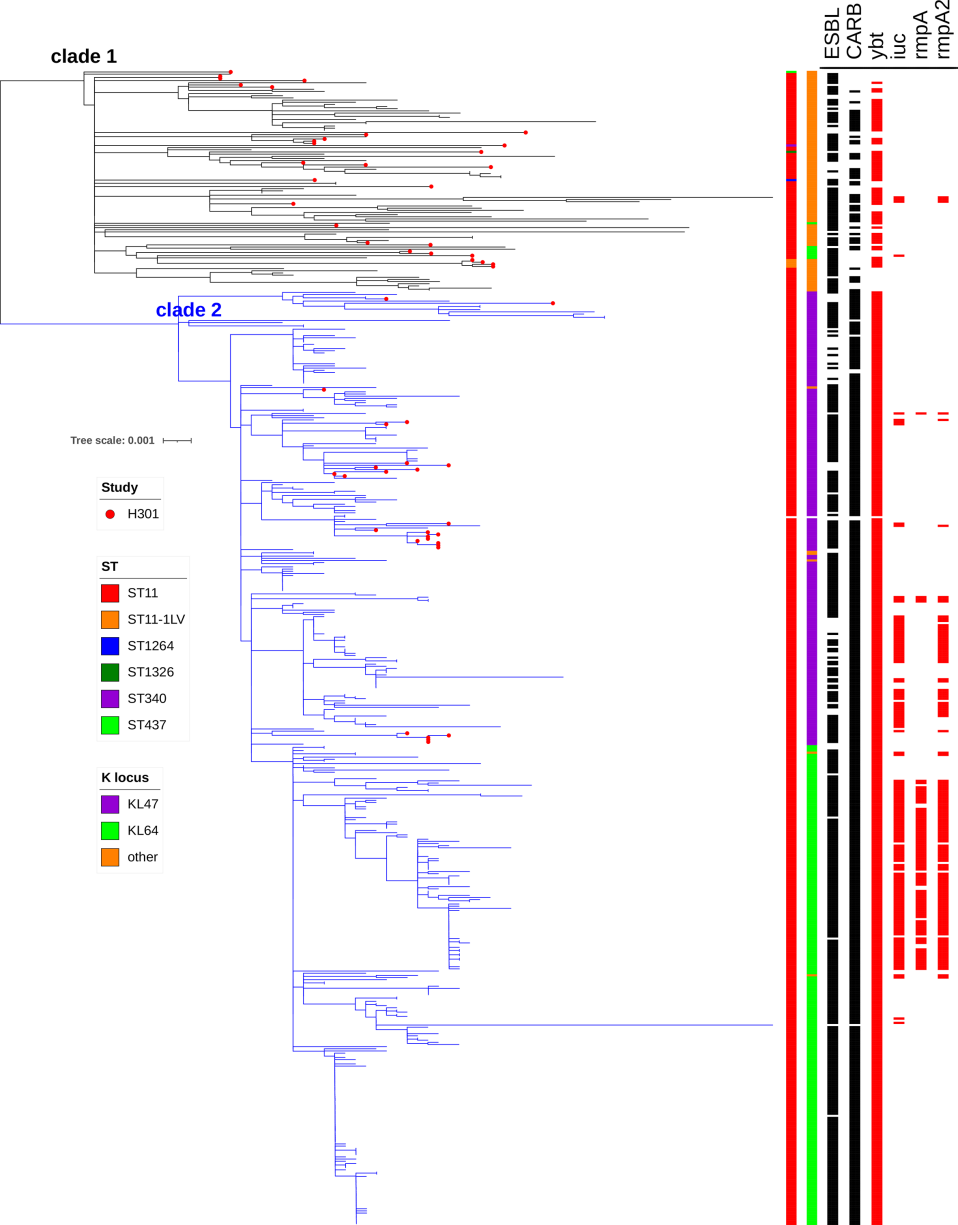
Phylogenetic analysis of CG258 strains, including 56 strains from this study, indicated by red dots on the tree leaves, and 479 strains from previous studies [18–20, 45]. The different STs and K loci are indicated by coloured boxes on the first and second metadata columns, respectively. The presence/absence of AMR and virulence genes is indicated by full/empty boxes, in black and red metadata columns, respectively. CARB, carbapenemase-encoding genes.

The phylogenetic analysis of CG258 genomes from this study together with publicly available CG258 genomes revealed the presence of two major clades as has been reported previously [19, 45] (Fig. 5). Clade 1 consisted of genomes obtained from several global countries and comprised several different STs and K loci, while clade 2 consisted of genomes of ST11 and K loci of type KL47, KL64 or KL31 originating from China. Strains belonging to ST11/KL47 from our study were all located within clade 2 and they formed distinct clusters, which is consistent with multiple independent introductions of this clone to the H301 hospital, followed by occasional transmission within the hospital. One putative transmission cluster involved four patients admitted at the surgical ICU in April 2015, from which eight isolates were obtained with a mean of three core pairwise SNPs (min 0, max 6).

Conversely to ST11/KL47 Kp, ST11/KL64 Kp strains from this study did not cluster within clade 2 together with previously reported ST11/KL64 Kp strains, but they were located within clade 1. ST11/KL64 Kp has recently gained attention due to its increasing prevalence in China, strong association with *bla*_KPC-2_ and enhanced mortality compared to ST11/KL47 strains [19, 25]. Previous genomic analyses revealed that this CG258 sub-clone descended from an ST11/KL47 ancestor after recombination of the capsule locus genes around 2011 [19]. Our analysis of recombination sites revealed that the ST11/KL64 Kp strains from this study had two major regions of recombination, the capsule locus genes and the ICE*Kpn*HS11286-1 region [46](distinct from those reported previously). The three ST11/KL64 kp strains in our collection were isolated in 2006 and 2007, they lacked the *bla*_KPC-2_ gene and the *ybt* locus, which is common to ST11/K64 within clade 2. Together these findings suggest a different evolutionary origin of ST11-KL64 Kp strains from this study compared to the emerging and broadly disseminating sub-clone described by Zhou *et al* [19].

We detected three cases of genotypic convergence of MDR and hypervirulence-associated genes within our CG258 population. The first strain (K7069) was isolated in 2007, belonged to ST11/KL64 and carried a pLVPK-like plasmid containing *iuc1* and *bla*_CTX-M-3_, *armA* and several other AMR genes. The other two strains belonged to ST11/KL47 (K63 and K920), carried a pLVPK-like plasmid containing *iuc1* and a truncated *rmpA2*, *bla*_CTX-M-65_ and *bla*_KPC-2_ genes, and were isolated in 2015 and 2016, respectively. The *iuc1*-positive ST11 genomes from this study appear to represent independent acquisitions of the virulence plasmid from the previous reports in the literature (Fig. 5). However, the tree suggests that for strain K920 the acquisition occurred in a shared ancestor with strain WCHKP020030 (assembly no. GCA_003038215.3, unpublished) isolated from a patient in Chengdu, China, in 2016. The complete WCHKP020030 genome was used as the reference for mapping the reads of strain K920, as the two genomes differed by only 15 core SNPs. The analysis supported the hypothesis that the *iuc1* locus in K920 was located on a highly similar replicon to that of WCHKP020030; a ~290 Kbp IncFIB plasmid containing a ~70 Kbp pLVPK-like region and several AMR genes (*bla*_OXA-1_, *sul1*, *arr-3*, *catB3* and *aac(6′)-Ib-cr5*).

### Phylogenetic analysis of the hypervirulent CG23

**Fig. 6.**
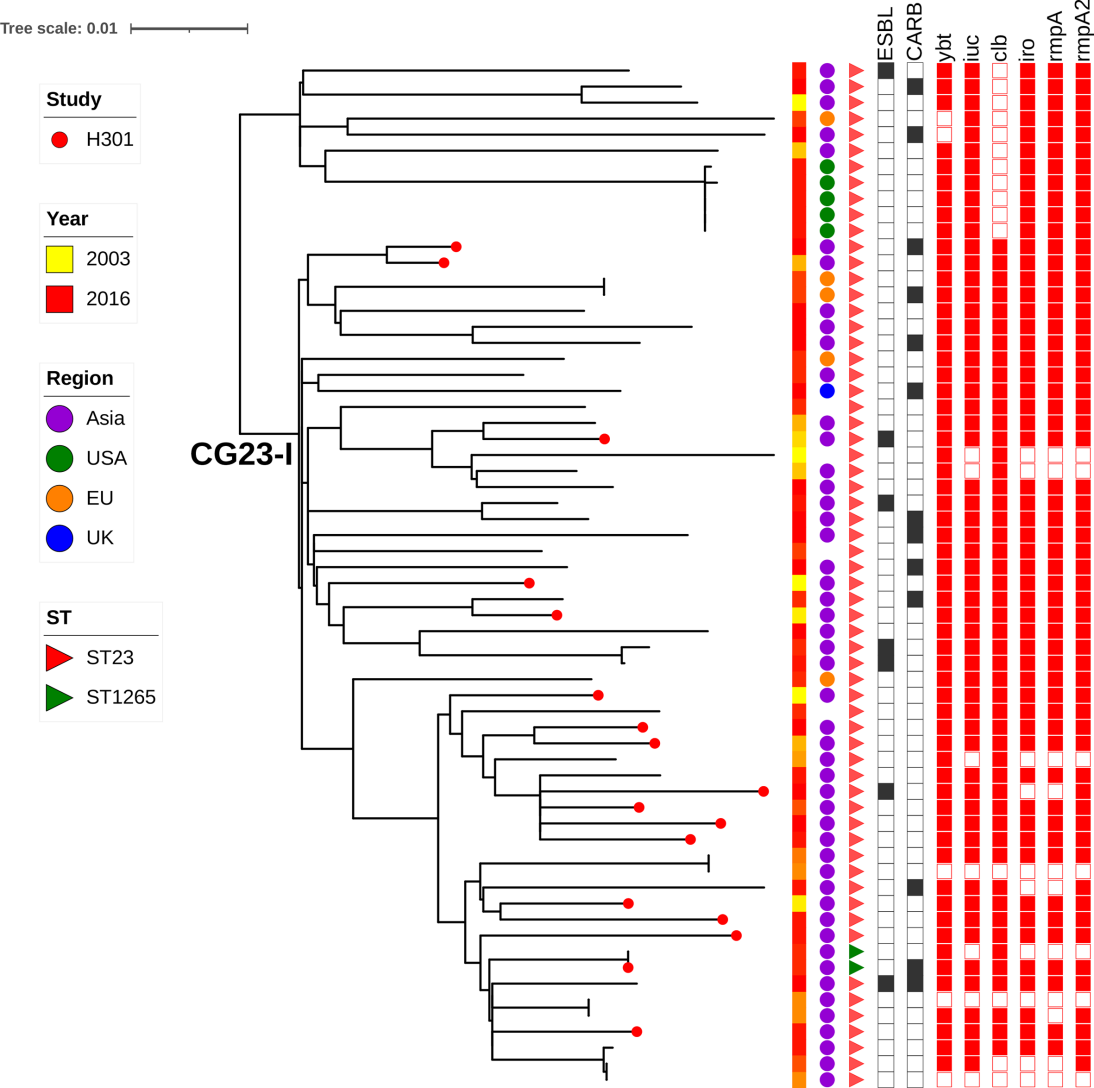
Comparative genomics of CG23 strains from this study, indicated by red dots on the tree leaves, also including publicly available genomes (*N*=47). The year of isolation, region of isolation and ST are indicated by the first three metadata columns, respectively. The presence/absence of AMR and virulence genes is indicated by full/empty boxes, in black and red metadata columns, respectively. CARB, carbapenemase-encoding genes.

A total of 17 non-duplicate CG23 strains were present in the broad sample collection, and phylogenetic analysis showed all belonged to the globally disseminated CG23-I sublineage (Fig. 6) [47]. All strains belonged to ST23, except strain K7159, which belonged to ST1265. This ST shares six MLST genes with ST23, differing only for allele *phoE*. ST1265 was first described in Beijing in 2010, associated with KL1 cps type, *rmpA* and a negative string test [48]. Our recombination analysis revealed that strain K7159 had a ~750 Kbp recombinant region, which contained the *phoE* gene. Genomic comparison revealed that this region likely originated from an ST35 genome (strain ABFQB, accession no. CP036438.1 and strain RJY9645, accession no. CP041353.2), suggesting that ST1265 is a hybrid of ST23 and ST35.

Fig. 6 shows the phylogenetic relationship between CG23 genomes from this study and publicly available CG23 genomes. Conversely to what was observed for ST11/KL47, we did not observe clustering of genomes, indicating multiple independent introductions in the hospital without further spread.

All of the CG23 genomes in our collection contained the KL1 capsular locus, the chromosomally encoded *ybt1* embedded in ICE*kp10* and the colibactin locus *clb2,* which is characteristic of the CG23-I sub-lineage. The virulence plasmid with IncFIB(K) and IncHI1B replicons was observed in all the strains, containing *iuc1*, *iro1*, *rmpA* and *rmpA2* in most instances (Fig. 4). Additionally, four genomes were detected with acquired AMR genes, including ST1265 isolate K7159. Mapping the reads of genome K7159 against the completed genome of the closely related 11420 strain (differed by six SNPs, isolated in Beijing in 2014 [49]) supported the presence of an IncFIB/IncHI1B pLVPK-like plasmid of size 229 796 bp containing *iuc1*, iro1 and *rmpA* and a separate KPC-2 plasmid of size 81180 bp, containing the replicon IncN without additional AMR genes.

Three additional genotypic convergences of MDR and hypervirulence-associated genes were also observed. Strains K931 and K862 both carried a ~50 Kbp IncN plasmid, as revealed by the manual inspection of the assembly graph, similar to pIMP-HZ1 (KU886034.1) described in an IMP-4-producing *Enterobacteriaceae* from China [50]. While K862 carried a plasmid identical to pIMP-HZ1, the IncN plasmid from strain K931 had *bla*_CTX-M-3_ and *bla*_TEM-1_ replacing the *bla*_IMP-4_ gene. Strain K7046 had a plasmid identical to pCTX-M-3 (AF550415) described in *C. freundii* in Poland [51]. It is a ~90 Kbp, IncL/M plasmid carrying *bla*_CTX-M-3_, *armA* and several other AMR genes *[bla*_TEM-1_, *aac(3)-IId*, *mph(E)*, *msr(E)*, *sul1*, *aadA2* and *dfrA12*].

### Global comparison of ST383: an emerging high-risk clone

**Fig. 7.**
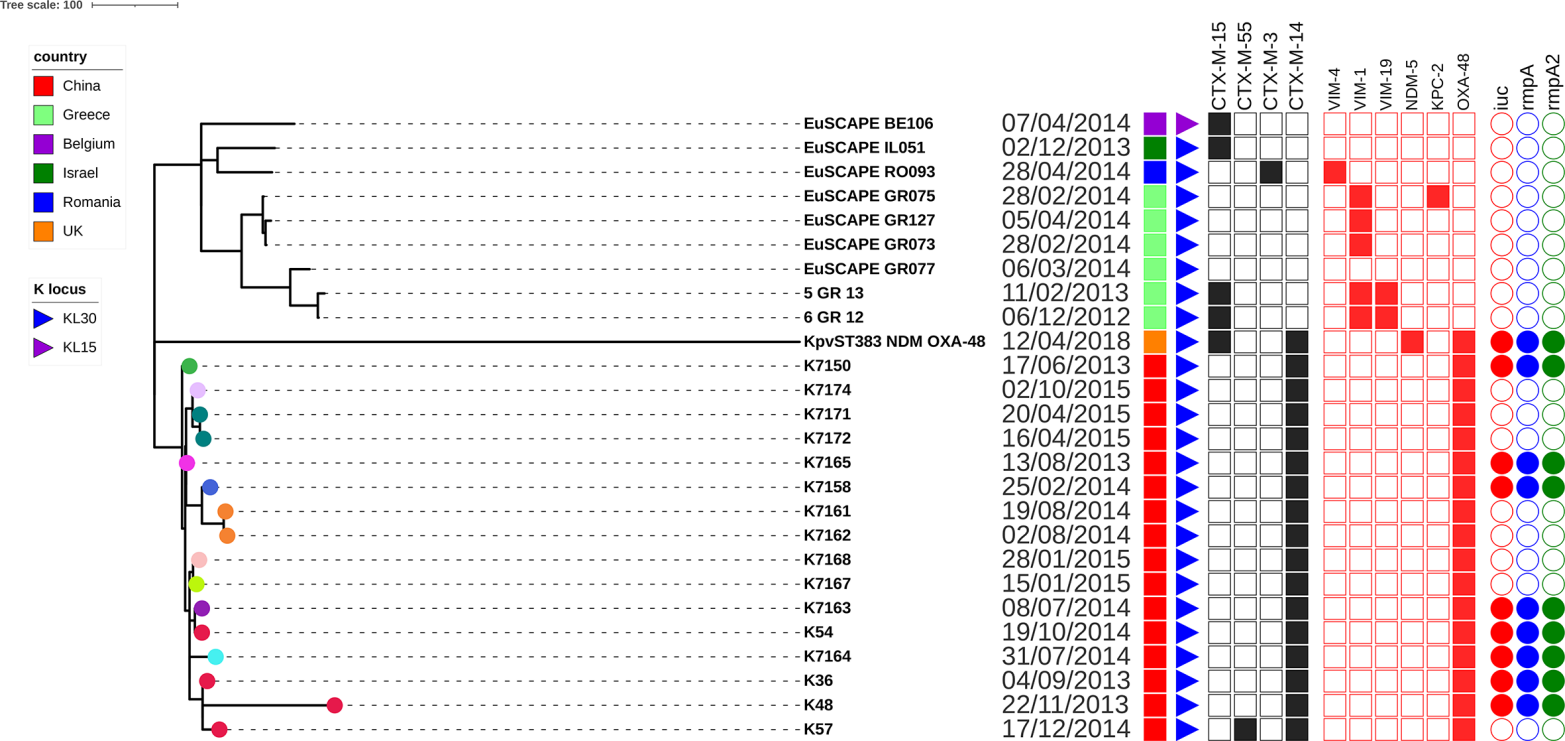
Phylogenetic tree of ST383 genomes from this study in comparison with publicly available ST383 genomes. Coloured leaves indicate different patient origin. The isolation date is showed, together with country of origin and K locus type, and the presence/absence of genes encoding for ESBLs, carbapenemases or virulence factors is represented by filled or empty shapes, respectively.

We performed further analyses of strains belonging to ST383 as we found several carrying both carbapenem-resistance and hypervirulence-associated genes. Fig. 7 shows the phylogenetic relatedness of the ST383 strains obtained from our broad sample collection (*N*=16) together with publicly available ST383 genomes. Only ten genomes were available, with most of them originating from Greece. Strain KpvST383_NDM_OXA-48 (CP034200) from the UK had a complete genome and it was used as the reference for the phylogeny [52]. Genomic relatedness showed strains from Europe clustering together, the strain from the UK apart from the rest of the tree, and the Chinese strains from this study clustering together. Overall, a mean of 158 core pairwise SNPs was observed (min: 4; max: 627; median: 157), which decreased to 53 (min: 4; max: 182; median: 40) if we only consider the strains from China. Two different K loci were observed, with the strain from Belgium carrying KL15 and all other strains carrying KL30. Gubbins analysis revealed that the capsular polysaccharide genes represented the major recombinant region, as is characteristic of MDR *K. pneumoniae* clones [53]. A second recombination concerned a ~12 Kbp region consisting of mercury resistance genes and several transposases. No other major recombinations were observed. Several carbapenemase-encoding genes were observed, comprising the major clinically relevant KPC, OXA-48, NDM and VIM types, with two strains coharbouring two different carbapenemase genes. All strains from China carried the *bla*_OXA-48_ gene and had an IncL/M plasmid replicon. ESBL-encoding genes were *bla*_CTX-M-14_, observed in all strains from China, and strain K57 additionally had *bla*_CTX-M-55_.

Concerning virulence factors, yersiniabactin-encoding genes were not observed overall. Conversely, the hypervirulent pLVPK-like plasmid was observed in some strains from China and in the strain from the UK. Although it was not possible to reconstruct the full virulence plasmids from our short-read sequence data, we detected *iuc1* on a contig that matches a 45 kb region of pLVPK and also carries *rmpA* and *rmpA2*.

## Discussion

This study aimed to understand the population structure of *K. pneumoniae* within the People’s Liberation Army General Hospital (H301) in Beijing over a 15 year period. While several studies have investigated the genetic epidemiology of CR-Kp in China [19, 25, 26], this study represents the first longitudinal investigation focusing on the broad *K. pneumoniae* population from China.

In China, AMR is a major concern, especially for *K. pneumoniae *. Data from the China Antimicrobial Resistance Surveillance System (CARSS) revealed that the resistance rates of *K. pneumoniae* were on a rising trend and reached 34.5 and 8.7 % in 2016 to third-generation cephalosporins and carbapenems, respectively [54]. In line with such trends, our data indicated that *K. pneumoniae* resistance rates reached 50.8 and 8.5 % in 2016 for ceftazidime and imipenem, respectively, within H301. The rising ceftazidime resistance rate was mirrored by an increase in ESBL prevalence, encoded mainly by genes of the CTX-M type. Four clones were mostly associated with the carriage of ESBL-encoding genes, being ST307, ST383, ST15 and CG258. Alarmingly, emerging clones, such as ST307 and ST383, may further increase the ESBL prevalence over the coming years, as such clones are strongly associated with ESBL production (we detected 100 % prevalence for both STs) and are becoming dominant in some hospitals worldwide [55–57].

The carbapenem resistance rate was 0 % in the early time frame of the study period, and reached 8.5 % in the period 2014–2016 within H301. Such a rising trend was consistent with the emergence and expansion of carbapenemase-encoding strains, with *bla*_KPC-2_ representing two thirds of the carbapenemase-encoding genes. The main driver of carbapenem resistance was CG258, which represented ~60 % of the carbapenemase producers while only representing 14 % of the *K. pneumoniae* population. Consistently with previous studies, the major CG258 sub-clone was ST11/KL47/KPC-2, which also harboured *bla*_CTX-M-65_ in most instances [18–20].

Conversely to what was observed for most of the drugs, amikacin resistance rates did not change over time, as reported previously [58]. Consistent with this observation, the prevalence of 16S-RMTase-encoding genes did not change over the study period.

Siderophore gene acquisition was recently recognized as an important contributor to severe *K. pneumoniae* invasive disease [6, 59]. Lam *et al.* reported that *ybt* was present in 40.0 % of the CG258, 87.8 % of the hypervirulent CG23, and 32.2 % of the wider population. We observed *ybt* genes in 30.2 % of the wider population, 100 % of CG23 strains, and 62.5 % of CG258 strains. *ybt* prevalence within CG258 was significantly higher than previously reported [6], and this is due to the abundance of strains belonging to the ST11/KL47/KPC-2 sub-clone that also harbours *ybt9*. Alarmingly, two ST11/KL47/KPC-2 strains also carried a pLVPK-like hypervirulent plasmid containing *iuc*, which is considered a key genetic trait for hvKP [60, 61].

Strains belonging to ST11/KL47/KPC-2, including those carrying *iuc*, clustered within the recently described sub-clade 2 together with strains from China causing outbreaks, including the fatal outbreak resulting in five deaths in 2017 [18–20]. Retrospective studies have shown that ST11/KL47/KPC-2 carrying *iuc* emerged before 2015 and has since become detectable in several different geographic locations including multiple provinces in China, as well as Hong Kong and India, raising concerns that these strains have the potential for worldwide dissemination [62–64]. We also observed an ST11/KL64 strain harbouring the virulence plasmid and the ESBL-encoding gene *bla*_CTX-M-3_, which represents an independent emergence from the recently described ST11-KL64 clade 2 strains that are increasing in prevalence in China and are associated with increased mortality rates compared to their ST11/KL47 ancestor [19, 25]. Our ST11/KL64 strain was collected in 2007, several years before the estimated emergence of clade 2. Unlike the clade 2 strains, there is no evidence that the descendants of this clade 1 ST11/KL64 have been able to disseminate. Nevertheless, we argue that further genomic surveillance investigations should be alert on distinguishing these two clones.

Similar to previous reports, CG23-I was detected in our collection as the dominant hvKp clone. While it is usually susceptible to multiple antibiotics, we found some strains harbouring MDR plasmids encoding for ESBLs and carbapenemases. Moreover, we found a strain belonging to the recently described ST1265 and showed that it is a hybrid strain originating from an ST23 and an ST35, carrying the KL1 capsule locus, the virulence plasmid and a KPC-2 plasmid. Our isolate was highly similar to that described previously (six SNPs), which was shown to be as virulent as hvKp ST23 in the *G. mellonella* infection model [49]. Fortunately, unlike the convergent CG258 strains discussed above, there was little evidence that this convergent CG23 strain is able to disseminate.

Aside from the convergent CG258 and CG23 isolates, we detected MDR-virulence convergence in six additional STs, accounting for 5.5 % of our random sample collection, which is similar to a recent study of bloodstream infection isolates from South and Southeast Asia (7.3 % convergent isolates, with seven different STs observed) [65]. Like this previous study, we detected examples of MDR clones that have acquired a virulence plasmid as well as hypervirulent clones that have acquired one or more MDR plasmids. The majority of these isolates appeared to represent sporadic convergence events with limited to no dissemination of the resulting strains. However, notable exceptions were ST11/KL47 (discussed above) and ST383.

ST383 is an emerging clone that was first observed in Greek hospitals during 2009–2010 and coharbouring *bla*_VIM-4_, *bla*_KPC-2_ and *bla*_CMY-4_ β-lactamases [66]. Strains belonging to ST383 and carrying OXA-48 plasmids were previously described, with reports from the UK [67] and from China [68]. In the latter study, Guo *et al.* reported an outbreak caused by ST383 strains carrying a 70 Kb IncL/M OXA-48 plasmid. ST383 strains carrying acquired virulence genes were also observed in the UK, carrying the *iuc* and *rmpA/A2* genes together with carbapenemase-encoding genes of type *bla*_OXA-48_, sometimes in combination with *bla*_NDM_ [52, 69]. Our genome collection contained 16 ST383 isolates, all carrying the carbapenemase-encoding gene *bla*_OXA-48_ in addition to the *iuc1* virulence locus. The median pairwise SNP distances between isolates was 53 SNPs (range 4–182 SNPs) supporting local clonal expansion. Therefore, our data add to the growing body of evidence that ST383 Kp is an emerging public health concern [56], able to readily acquire carbapenemase-encoding genes of different types as well as genes associated with the hypervirulence phenotype. Tracking the evolution and distribution of such a clone is of major importance.

Overall, the whole-genome sequencing of a large number of clinical *K. pneumoniae* isolates, together with their associated phenotypes, can be considered as the major strength of this study. However, the fact that the isolate collection was spread over a 15 year period prevented statistical investigations of gene prevalence, such as AMR and virulence genes, over time. Therefore, either sequencing more isolates or reducing the time frame would have benefited our investigation. As a future perspective, long-read sequencing of some representative isolates may be helpful in resolving the complete structure of plasmids, and this would be interesting especially for the detection of novel MDR-hypervirulence hybrid plasmids.

In conclusion, this study represents the first genomic investigation of the broad *K. pneumoniae* population in China. We showed that such population is highly diverse, consisting of both known and emerging ESBL-, carbapenemase- and hypervirulence-associated clones. The combination of MDR and hypervirulence significantly reduces the antimicrobial options for treating the life-threatening infections caused by such strains and therefore represents a major and urgent challenge for clinical treatment, infection control and public health management [70]. Through the analysis of a broad sample of clinical *K. pneumoniae* isolates, our data supports, together with previous studies, the hypothesis that convergent MDR-virulent isolates may emerge at a significant pace in some parts of the world (including China, South and South East Asia) [19, 65]. There is therefore an urgent need for high-resolution genomic surveillance to detect novel convergent isolates, and rapidly distinguish the majority of sporadic strains from the minority that are able to spread.

## Supplementary Data

Supplementary material 2
