# Corrigendum: Genomic evolution and local epidemiology of *Klebsiella pneumoniae* from a major hospital in Beijing, China, over a 15 year period: dissemination of known and novel high-risk clones

**DOI:** 10.1099/mgen.0.000618

**Published:** 2021-08-18

**Authors:** Mattia Palmieri, Kelly L. Wyres, Caroline Mirande, Zhao Qiang, Ye Liyan, Chen Gang, Herman Goossens, Alex van Belkum, Luo Yan Ping

**Affiliations:** ^1^​ bioMérieux, Data Analytics Unit, La Balme Les Grottes, France; ^2^​ Department of Infectious Diseases, Central Clinical School, Monash University, Melbourne, Victoria, Australia; ^3^​ bioMérieux, R&D Microbiology, La Balme Les Grottes, France; ^4^​ Center for Clinical Laboratory Medicine, First Medical Center of Chinese PLA General Hospital, Beijing, PR China; ^5^​ Laboratory of Medical Microbiology, Vaccine and Infectious Disease Institute, University of Antwerp, Belgium

## Full-Text

In the published version of this article, the corresponding author was listed incorrectly. The corresponding author statement should have read as follows:

‘*Correspondence: Luo Yan Ping, ypluo301@aliyun.com’

Additionally, in the first paragraph of the discussion section some text and accompanying references were mistakenly omitted. The omitted references and corrected text with corresponding citations are detailed below:

‘This study aimed to understand the population structure of *K. pneumoniae* within the People’s Liberation Army General Hospital (H301) in Beijing over a 15 year period. While several studies have investigated the genetic epidemiology of CR-Kp in China in general [19, 25, 26] and in Hospital 301 in particular [1–6], this study represents the first longitudinal investigation focusing on the broad *K. pneumoniae* population from China.’

1. Yang J, Ye L, Guo L, Zhao Q, Chen R, *et al*. A nosocomial outbreak of KPC-2-producing *Klebsiella pneumoniae* in a Chinese hospital: dissemination of ST11 and emergence of ST37, ST392 and ST395. *Clin Microbiol Infect*. 2013;19(11):e509–15 .

2. Zhou G, Guo S, Luo Y, Ye L, Song Y, *et al*. NDM-1-producing strains, family *Enterobacteriaceae *, in hospital, Beijing, China. *Emerg Infect Dis*. 2014 ;20(2):340–2

3. Luo Y, Wang Y, Ye L, Yang J. Molecular epidemiology and virulence factors of pyogenic liver abscess causing *Klebsiella pneumoniae* in China. *Clin Microbiol Infect*. 2014;20(11):O818–24

4. Guo L, An J, Ma Y, Ye L, Luo Y, *et al*. Nosocomial outbreak of OXA-48-producing *Klebsiella pneumoniae* in a Chinese hospital: clonal transmission of ST147 and ST383. *PLoS One*. 2016;11(8):e0160754

5. An J, Guo L, Zhou L, Ma Y, Luo Y, *et al*. NDM-producing *Enterobacteriaceae* in a Chinese hospital, 2014-2015: identification of NDM-producing *Citrobacter werkmanii* and acquisition of blaNDM-1-carrying plasmid *in vivo* in a clinical *Escherichia coli* isolate. *J Med Microbiol*. 2016;65(11):1253–1259

6. Lai K, Ma Y, Guo L, An J, Ye L, *et al*. Molecular characterization of clinical IMP-producing *Klebsiella pneumoniae* isolates from a Chinese Tertiary hospital. *Ann Clin Microbiol Antimicrob*. 2017;16(1):42

The authors apologise for any inconvenience caused.

